# Prime Time or Lyme Time: A Case of Lyme Hepatitis in the Emergency Department

**DOI:** 10.7759/cureus.66713

**Published:** 2024-08-12

**Authors:** Megan E Weis, Danny Le, Timothy J Friel, Michelle N Carraro

**Affiliations:** 1 Department of Emergency and Hospital Medicine, Lehigh Valley Health Network/USF (University of South Florida) Morsani College of Medicine, Allentown, USA; 2 Department of Medicine, Lehigh Valley Health Network/USF (University of South Florida) Morsani College of Medicine, Allentown, USA

**Keywords:** emergency management, transaminitis, abnormal liver studies, erythema migrans, lyme disease

## Abstract

Lyme disease, a tick-borne illness, is caused by the spirochete *Borrelia burgdorferi*. Lyme disease commonly presents with the characteristic erythema migrans rash, fever, malaise, headache, and arthralgias. Some patients may have mild liver manifestations, including abnormal liver function tests (LFTs), hyperbilirubinemia, or granulomatous hepatitis. Significant LFT abnormalities and hepatitis in a case of Lyme disease are rare. Here, we present a case of Lyme hepatitis in the emergency department (ED) where the patient presented with classic Lyme symptoms and was also found to have markedly elevated aspartate transaminase (AST) and alanine transaminase (ALT), mild alkaline phosphatase (ALP) elevation, and mild hyperbilirubinemia.

## Introduction

Lyme disease is a tick-borne disease caused by the spirochete *Borrelia burgdorferi *[1]. It is transmitted by the *Ixodes *tick and is endemic to the northeastern and north-central United States [2]. Some symptomatic patients may not recall a tick bite, but 60% will present with the characteristic erythema migrans - a red, rounded rash that is well-defined with a central clearing that leaves a target or bull-eyed appearance [3]. This rash may expand and enlarge over days to weeks and multiply in disseminated infection [2]. Other symptoms may include fever, malaise, headache, and arthralgias [2,3]. Some patients, especially those with disseminated infection, have liver manifestations including abnormal liver function tests (LFTs) and hyperbilirubinemia [1]. Severe hyperbilirubinemia and granulomatous hepatitis are rarer occurrences [1]. In some cases, patients will have mild (defined as less than five times the upper limit of normal) elevation of LFTs such as aspartate transaminase (AST) and alanine transaminase (ALT), suggesting minor transaminitis [1,2,4]. This will occur even in the absence of underlying liver disease and will typically resolve with treatment [1]. Here, we present a case of a patient who presented to the emergency department (ED) with fever, fatigue, multiple bulls-eye rashes, and elevated liver function tests with a positive Lyme test.

## Case presentation

**Figure 1:**
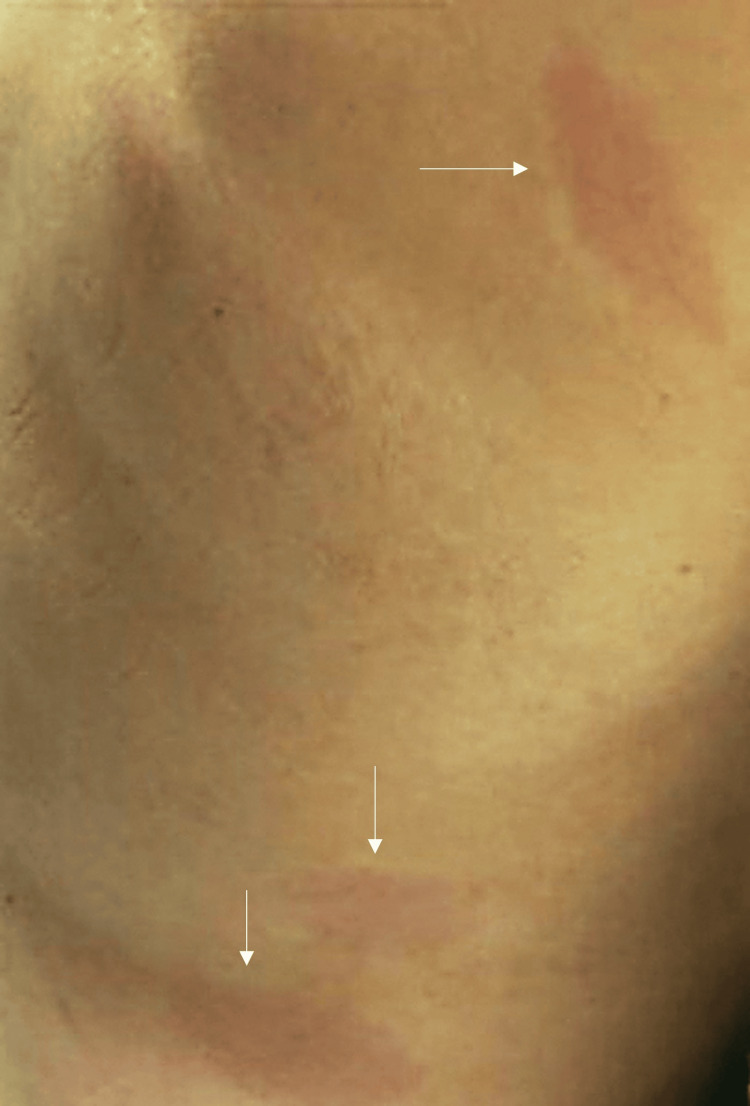
Erythema migrans rashes on the patient’s right side identified by arrows

A 56-year-old male presented to an ED in Pennsylvania with two weeks of generalized fatigue, fevers, and myalgias. His past medical history was only significant for tobacco use. Prior to this ED visit, he was seen at an urgent care where he was tested for Lyme and was negative. During that visit, he was given ibuprofen and advised to stay hydrated. Given the duration of his symptoms, he presented to the ED for further evaluation. He reported fevers with a max temperature of 102 °F. He also reported multiple lesions reminiscent of erythema migrans that appeared on his body about three days prior (Figure 1). Of note, the rashes were not present during his urgent care visit.

**Figure 2:**
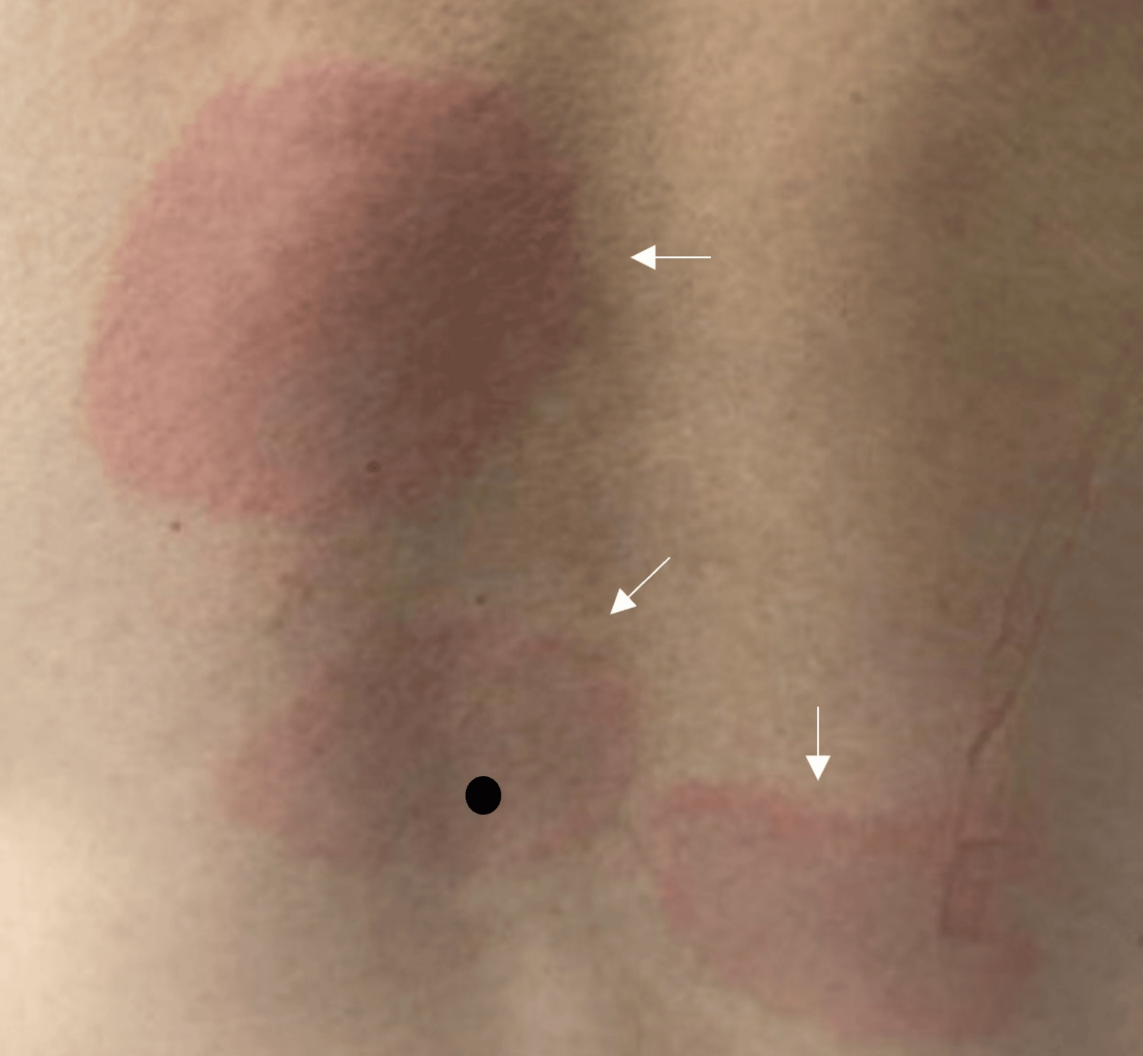
Bulls-eye rashes on the patient’s back identified by arrows

He denied any recent sick contacts, travel history, or notable tick removal. He was afebrile and hemodynamically stable. Physical exam was only significant for multiple lesions on his back and his legs (Figure 2).

His ED evaluation included blood work and imaging, revealing a leukocytosis at 14.4 thou/cmm (ref 4.0-10.5 thou/cmm) with neutrophil predominance at 11.9 thou/cmm (ref 1.8-7.8 thou/cmm). His LFTs were markedly elevated when compared to his last blood work three years prior. His AST was 207 U/L (ref < 41 U/L), ALT was 603 U/L (ref < 56 U/L), and ALP was 368 U/L (ref 35-120 U/L). His total bilirubin was slightly elevated at 1.1 mg/dL (ref 0.2 - 1.0 mg/dL). Given the hepatic derangements, infectious disease was consulted and recommended observation in the hospital as well as further workup for other tick-borne illnesses and hepatitis. He was given a dose of oral doxycycline 100 mg before admission. During his hospital stay, testing for Ehrlichia, Anaplasma, Babesia, and hepatitis B and C were negative. Lyme titers that were ordered in the ED came back positive. The following day, his repeat blood work showed improvement in his LFTs, AST was 141, ALT 457, and ALP 330 U/L, and he was discharged home on doxycycline 100 mg twice daily for 21 days. On a follow-up appointment one month later, his LFTs normalized to AST 17, ALT 19, and ALP 68 U/L and his rash had resolved.

## Discussion

This case demonstrates an additional consideration when evaluating patients with abnormal liver studies in the ED. This patient presented with vague symptoms of fever and malaise for two weeks before developing the classic erythema migrans rash. At that time, he was found to have a significant elevation in his LFTs. The differential diagnosis of patients with LFT elevation and rash is quite broad and includes but is not limited to other significant illnesses such as syphilitic hepatitis (maculopapular rash) [5], chronic liver disease (spider nevi, palmar erythema, dry itchy rashes) [6], and drug-induced liver injury [7]. While our case was in an endemic area for Lyme disease (Pennsylvania), evaluation of underlying liver disease was recommended because the elevated LFTs were more significant than the mild transaminitis expected in Lyme disease. One study showed only 14% of patients with Lyme had liver tests over two times the limit of normal [8]. Additionally, this patient had a slightly abnormal bilirubin level. Only 3% of Lyme disease patients were found to have abnormal bilirubin labs in at least two different studies of liver function in Lyme disease [1]. Since all the other infectious workups were negative for our patient, the laboratory derangements can likely be attributed to Lyme disease. This is also supported by the complete resolution of his abnormal lab levels one month later after treatment with doxycycline.

## Conclusions

This case report describes a patient who presented to the ED with fever, fatigue, multiple bulls-eye rashes, and elevated liver function tests with a positive Lyme test. Some patients may present with mild liver manifestations, including abnormal liver function tests (LFTs), hyperbilirubinemia, or granulomatous hepatitis. In cases of otherwise unexplained elevations in AST, ALT, ALP, and/or bilirubin, Lyme disease should be considered in the differential diagnosis in the ED. It should be considered especially in regions of the US with a high incidence of this infection, which further emphasizes the need to evaluate travel history in patients.
